# Individual and cumulative benefits of making body armour and chemical & biological protective gloves, respirator and overboots from moisture vapour permeable materials

**DOI:** 10.1186/2046-7648-4-S1-A96

**Published:** 2015-09-14

**Authors:** Christie Garson, Michael Dennis, Michael J Tipton, James R House

**Affiliations:** 1 Extreme Environments Laboratory, Sport and Exercise Science, University of Portsmouth, UK; 2 Defence Science and Technology Laboratory, Salisbury, UK

## Introduction

Some chemical & biological (CB) personal protective equipment, such as gloves, overboots and respirators, and other equipment such as body armour (BA) are made from moisture vapour impermeable (MVIP) materials, which increase insulation and impede evaporative cooling, thereby increasing the thermal burden. The aim of this study was to quantify the thermal burden imposed by each individual MVIP CB protective item and that imposed by BA.

## Methods

Following a favourable ethical opinion 12 males volunteered for this experiment. The study was a five-condition, repeated measures design with stepping at a light intensity ($\overset{\cdot }{\text{V}}{\text{O}}_{\text{2}}$ 13.5 mL.kg^-1^.min^-1^), interspersed with 20-minute rest periods in a hot and dry environment (40.5 **°**C and 20 % rh) for a maximum of 170 minutes, the last hour of which was continuous work. Actual body armour was not used and instead a MVIP BA liner (BAL) (mass of 170 g) was used to mimic the impermeability of BA but without the mass. Conditions varied in which combinations of the MVIP items (respirator [R], BAL [A], gloves [G] and overboots [O]) were removed, with the mass of that item being substituted (at the area from which the item was removed), thereby simulating making the item 100 % MVP but without altering the metabolic cost imposed by carrying the item. All conditions included wearing a chemical protective suit (S) and were: Control (SOGAR), SOGA (no R), SOG (no A or R), SO (no G, A or R), and S (Suit only). Conditions were compared against adjacent conditions (*i.e*. SOGAR *vs*. SOGA; SOGA *vs*. SOG; SOG *vs*. SO and SO *vs*. S) to quantify any reduction to thermoregulatory strain when removing R, A, G and O separately. All statistical analyses were conducted on Prism 6 (GraphPad). It was hypothesised that thermal strain would be improved by this order: BAL>G>R>O, with the benefits of R being predominantly perceptual.

## Results

Removing BAL resulted in an improvement to the sweat production/evaporation ratio by 17.3 % (p < 0.0001) culminating in 9 participants completing the final 60 minutes of exercise compared to 4, 10 and 11 participants when R, G or O were removed respectively. Removing BAL also resulted in an attenuated increase in rectal temperature (ΔT_re_) from one hour into the protocol, with a maximum attenuation of 0.27 °C (p < 0.0001). The change in mean body temperature was significantly reduced from as early as 40 minutes when BAL was removed with a maximum reduction of 0.31 **°**C (p < 0.0001). Removing BAL also significantly attenuated heart rate (HR) throughout the protocol by a maximum of 11 beats.min^-1 ^(p < 0.001) and resulted in a reduction of 22 % (p < 0.0001) to the physiological strain index (PSI). Perceptually, removing the BAL also improved measures of thermal sensation (TS) during exercise (p < 0.05) and thermal comfort (TC) during rest (p < 0.05). Removing G resulted in a 7.9 % improvement to the sweat production/evaporation ratio thereby reducing thermoregulatory strain with the greatest attenuation to T_re _of 0.37 **°**C at the end of the protocol (p < 0.0001). Mean skin temperature was attenuated by a maximum of 0.42 **°**C (p < 0.001). Removing G lowered HR during rest and improved perceptual measures: RPE (p < 0.0001), TS (p < 0.01) and TC (p < 0.01). The PSI was by improved by 13.1 % during the final work period (p < 0.001) when R was removed. Perceptually, removing R resulted in improved ratings of TS during the final work (12 %; p < 0.05) and rest periods (14 %; p < 0.01) as well as improved TC in the final rest period (14 %; p < 0.001). Removing O resulted in the least reduction to thermoregulatory strain.

## Conclusion

Improving the permeability of body armour and to a lesser degree CB gloves would be of significant physiological and perceptual benefit to the warfighter. Improving the permeability of the respirator would improve perceptual responses more so than physiological responses and we therefore accept our hypothesis. Improving the permeability of the CB overboots offers little thermal or perceptual benefit to the warfighter. These are ordered as per our hypothesis, which consequently we accept.

